# Comparison of a tridimensional cephalometric analysis performed on 3T-MRI compared with CBCT: a pilot study in adults

**DOI:** 10.1186/s40510-019-0293-x

**Published:** 2019-10-21

**Authors:** Cinzia Maspero, Andrea Abate, Francesca Bellincioni, Davide Cavagnetto, Valentina Lanteri, Antonella Costa, Marco Farronato

**Affiliations:** 1 Department of Orthodontics, UOC Maxillofacial and Dental Surgery, Fondazione IRCCS Ca’ Granda Ospedale Maggiore Policlinico, 20142 Milan, Italy; 2 University of Milan, Milan, Italy

**Keywords:** Tridimensional cephalometric analysis, Magnetic resonance imaging, Cone-beam computed tomography, Orthodontic diagnosis

## Abstract

**Objective:**

Since the introduction of cone-beam computed tomography (CBCT) in dentistry, this technology has enabled distortion-free three-dimensional cephalometric analysis for orthodontic and orthognathic surgery diagnosis. However, CBCT is associated with significantly higher radiation exposure than traditional routine bidimensional examinations for orthodontic diagnosis, although low-dose protocols have markedly reduced radiation exposure over time.

The objective of this preliminary feasibility study is to compare the accuracy and diagnostic capabilities of an already-validated three-dimensional cephalometric analysis on CBCT to those of an analysis on 3-T magnetic resonance imaging (3T-MRI) to assess whether the latter can deliver a comparable quality of information while avoiding radiation exposure.

**Materials and methods:**

In order to test the feasibility of three-dimensional cephalometry on 3T-MRI, 18 subjects (4 male; 14 female) with mean age 37.8 ± SD 10.2, who had undergone both maxillofacial CBCT and maxillofacial 3T-MRI for various purposes within 1 month, were selected from the archive of the Department of Dentistry and Maxillofacial Surgery of Fondazione Ospedale Policlinico Maggiore, IRCCS, Milano, Italy.

A three-dimensional cephalometric analysis composed of ten midsagittal and four bilateral landmarks and 24 measurements (11 angular, 13 linear) was performed on both scans using Mimics Research® v. 17.0 (NV, Technologielaan 15, 3001 Leuven, Belgium). Cephalometric analysis was performed twice by two independent orthodontists for each scan, and each orthodontist repeated the measurements 3 weeks later. Statistical analysis was performed with SPSS® 20.00 for Windows (IBM® Corporation, Sommers, NY, USA). A Bland-Altman test for each cephalometric value was performed to assess the agreement between the procedures. The intraclass correlation coefficient (ICC) was used to assess interobserver and intraobserver reliability. The coefficient of variation was used to evaluate precision.

**Results:**

Both procedures showed good reliability, with mean intraobserver ICCs of 0.977/0.971 for CBCT and 0.881/0.912 for MRI. The average interobserver ICCs were 0.965 for CBCT and 0.833 for MRI. A Bland-Altman analysis for the cephalometric tracing revealed a similar range of agreement between the two modalities; the bias range (mean ± SD) was − 0.25–0.66 mm (0.174 ± 0.31) for distances and − 0.41–0.54° (0.12 ± 0.33) for angles.

**Conclusions:**

Within the main limitation of this pilot study, that is, the small sample, it is possible to state that cephalometric measurements on 3T-MRI seem to possess adequate reliability and repeatability and that they show satisfying agreement with values measured on CBCTs. An MRI examination does not expose patients to ionizing radiation and could provide an alternative to CBCT for three-dimensional cephalometrics in the future.

## Introduction

Cephalometric tracings on lateral radiographs have remained the diagnostic method of choice in orthodontics since their appearance in the 1930s. However, diagnosis with 2D cephalometric radiographs has limitations due to bidimensional flattening, variable magnification of facial bones, overlapping of different structures, diagnostic reliability dependent on correct head position when taking the radiograph, and the need for additional teleradiographs in postero-anterior and axial projection to evaluate symmetry [1–4].

After the introduction of cone-beam computed tomography (CBCT) in dentistry in 1998, enabling the use of a lower radiation dose than multislice computed tomography (MSCT), several cephalometric three-dimensional tracings were developed [5]. The advent of 3D imaging technology and 3D software made it possible to visualize, study, and evaluate all three dimensions of the craniofacial structure with 3D analysis. However, to date, the role of magnetic resonance imaging (MRI) in 3D cephalometry has not been thoroughly studied because the examination time should be kept as short as possible and because the procedure needs to be well tolerated.

Measurements taken on CBCT have been demonstrated to be accurate and reliable by many studies [6–9].

Radiation exposure from new-generation CBCT, despite continuous reduction, is still significantly higher compared to conventional bidimensional radiographs [10], and therefore, indications are restricted to complex cases whose benefits from three-dimensional information justify the increased radiation exposure according to ALARA (as low as reasonably achievable) and ALADA (as low as diagnostically acceptable) principles [11]. Cone-beam computed tomography (CBCT) is now used for orthodontic diagnosis and treatment planning for conditions such as severe asymmetries, impaction of one or more teeth, and craniomaxillofacial malformation [11–13].

The developmental process of MRI was similar to that of CT. Although initially not suitable for hard tissue imaging because of its poor performance in recording details of mineralized tissues, the development of high-field scanners, dedicated coil systems [14–16] and application-optimized sequences [17, 18], and an increased field strength [19] led to substantial improvement of its detailed definition, which allowed us to visualize dental and periodontal structures. In particular, magnetic resonance imaging (MRI) is becoming a promising modality for cephalometric analysis through new 3D sequences with high spatial resolution as 3-T high-field MRI (3T-MRI).

A 3T-MRI scanner generates a magnetic field that is twice the strength of conventional 1.5-T machines and yields exceptional anatomic detail.

A 3-T scanner provides a stronger signal, higher resolution, higher sensitivity, shorter imaging times, and higher reliability than a 1.5-T scanner. These qualities allow the maximization of patient comfort and the enhancement of diagnostic capacity and accuracy [20]. Current 3T-MR systems enable morphological investigation with high spatial, temporal, and contrast resolution (essential for diagnosis), enhancing the diagnostic power of routine MRI in terms of sensitivity and specificity both in clinical practice and in applied research purposes. It lowers the risk of distorted images, thus eliminating the need for repeated scans [20].

MRI examinations are commonly requested in dentistry for the evaluation of soft tissue components of the temporomandibular joint and should precede cone-beam CT for cases in which the diagnosis of soft tissue pathology is a concern [21, 22]. MRI can be employed in the diagnosis and treatment planning of implants, jaw lesions, temporomandibular joints disorder (TMD), orthodontic treatment, and endodontic treatment to obtain a better prognosis [23]. In two studies, scientists compared 2D lateral cephalometric radiographs with sagittal MRI images [24, 25].

MRI may thus become a valid alternative diagnostic because it can reduce the radiation dose delivered to patients while obtaining similar information on CBCT scans, and it is also able to overcome the abovementioned limitations of bidimensional teleradiographs while adopting a radiation-free examination, thus obtaining a double advantage.

Consequently, we used an efficient 3D craniofacial analysis, and few landmarks are needed to identify the involved structures quickly and reliably [26] and with a short MRI scanning time.

The aim of the present study was to compare previously published three-dimensional cephalometric analysis for CBCT with the same analysis performed on 3T-MRI [26].

## Materials and methods

### Ethics and funding

Since our study design is retrospective, as all evaluations have been made on CBCT and MRI scans from the archive of the Department of Dentistry and Maxillofacial surgery of Fondazione IRCCS Cà Granda Ospedale Maggiore Policlinico, Milano, Italy, no ethical approval was gained [27, 28]. All patients whose reports were included in this study gave informed consent to undergo the examination and to eventually make their examination available for research purposes. The study protocol was approved by the appropriate institutional review board (IRB) within the research project of the year 2018 O.U.N. 420/425 of Fondazione IRCCS Cà Granda Ospedale Maggiore Policlinico, Milano.

### Types of participants

A sample of 18 Caucasian fully grown subjects (4 male; 14 female) with mean age 37.8 SD ± 10.2 was included in this study. Each of them received both a maxillofacial CBCT and a maxillofacial 3-T high-field MRI within 1 month for various reasons, mainly diagnosis and treatment planning of gnathological interventions and gnathological evaluation prior to orthognathic surgery in symptomatic patients. During this period of time, patients did not undergo any orthodontic or dental treatments.

The exclusion criteria were severe facial asymmetry, missing permanent incisors, and insufficient image quality of CBCT or MRI.

### MRI examinations

MRI scans were performed on all subjects using 3.0 T X series Philips Achieva system (Philips Healthcare, Best, Netherlands) at the Neuroradiology Department of Fondazione IRCCS Cà Granda, Ospedale Maggiore Policlinico, Milano, Italy.

T2-weighted images were considered because of their better contrast between soft and hard tissues and their lower total scan duration of 5 min and 27 s in turbo mode Technique SE, which allows minor motion artifacts to be present. The field of view covered all relevant cephalometric landmarks.

The parameters of the used MRI sequence are as follows: repeat time (TR) 2500 ms, echo time (TE) 280 ms, 1 NEX, ETL 65, bandwidth 255 Hz/pixel flip angle = 90°, field of view (FOV) 240 × 240 × 180 mm, voxel size (reconstructed voxel) 0.49 × 0.49 × 0.50 mm, section thickness 0.49 mm, and time of acquisition 5′27″.

### CBCT examinations

CBCT for all patients was obtained using the iCAT CLASSIC® cone-beam dental-imaging system (Imaging Sciences International, Hatfield, Pa 19440).

The parameters of the CBCT acquisition protocol were as follows: 4 mm slice thickness, a 170 × 230 mm field of view (FOV), a 20-s scan time, a 0.49 × 0.49 × 0.5 mm voxel size, 120 kVp, and 3–8 mA.

### Data processing and cephalometric analysis of CBCT and MRI

The processed volumetric data were exported by a radiologist (AC) as Digital Imaging and Communications in Medicine (DICOM 3) data set.

DICOM files were reconstructed into 3D images with Mimics Research® software v.17.0 (NV, Technologielaan 15, 3001 Leuven, Belgium) where three-dimensional cephalometry was performed [28].

Cephalometric analysis was performed on each scan by two orthodontists experienced in 3D dental cephalometry (observer I, FB; observer II, MF), who repeated the examinations after 3 weeks. Observers were blinded to patients’ identities.

First, three planes of reference have been created: midsagittal plane passing through Ba, S, and N; axial plane passing through S, N, and perpendicular to the midsagittal plane; and a coronal plane passing through S and perpendicular to the other two planes.

**Fig. 1:**
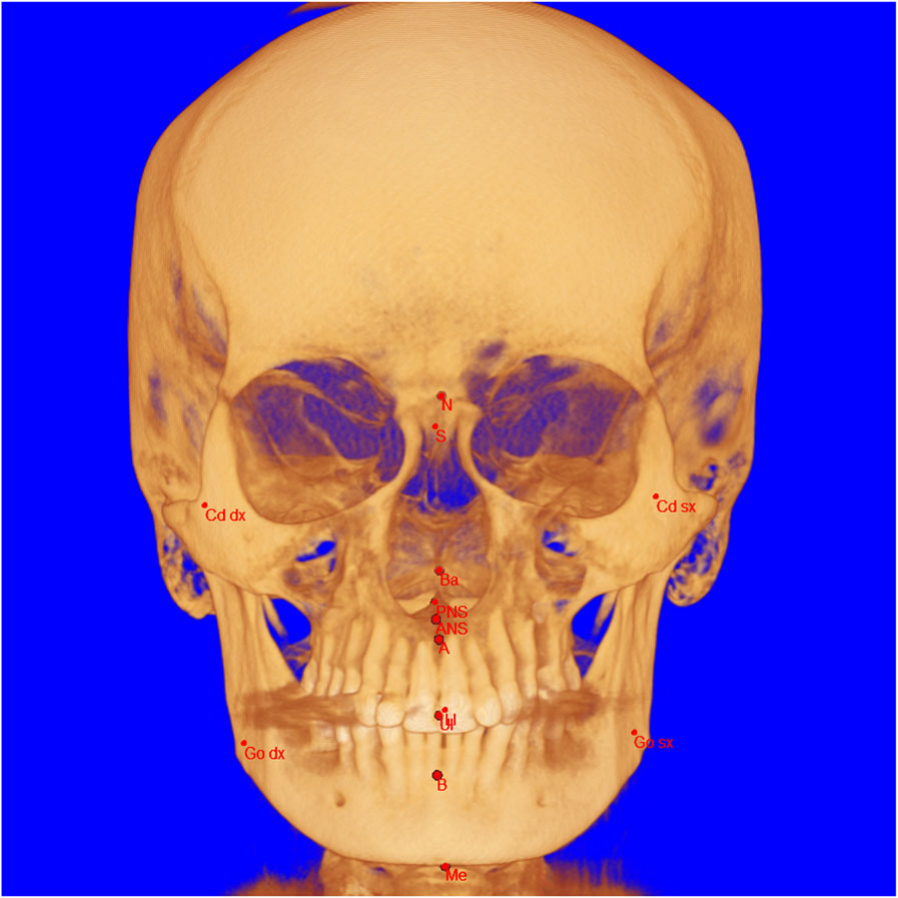
Cephalometric landmarks used in the present study. A total of 10 midsagittal and 8 bilateral landmarks were included in the cephalometric analysis: A = point A (most concave point of anterior maxilla); B = point B (most concave point of mandibular symphysis); ANS = anterior nasal spine; PNS = posterior nasal spine; Go = gonion; Ba = basion; S = sella; N = nasion; Cd = condylion; Go = gonion; LI = lower incisor; Me = menton; UI = upper incisor; Li = lower incisor; L/R = left/right

**Fig. 2:**
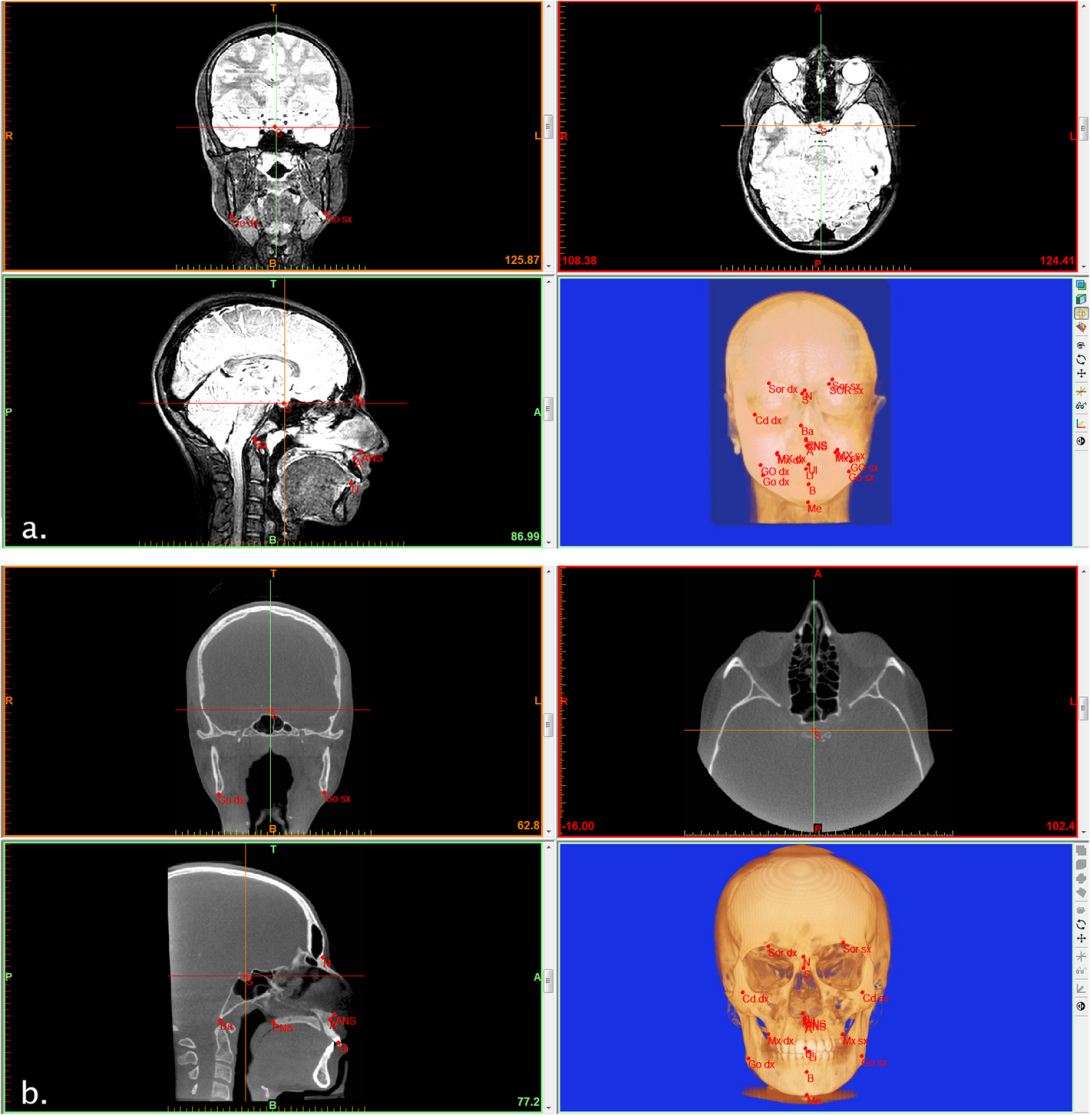
Multiplanar reconstruction and 3D reconstruction views after landmark identification. **a** Snapshot of software interface, showing 3T-MRI cephalometric landmarks on axial, coronal, and sagittal sections and on 3D volumetric rendering. **b** Snapshot of the software interface, showing CBCT cephalometric landmarks on axial, coronal, and sagittal sections and on 3D volumetric rendering

Ten midsagittal and four lateral symmetrical landmarks (Fig. 1) were identified in CBCT and 3T-MRI axial, coronal, and sagittal sections. The landmark position of each point was then checked on 3D volumetric rendering generated by the program (Fig. 2).

Each cephalometric report provided 13 linear and 11 angular measurements (Table 1) [26].

**Table 1:** Definition of the cephalometric measurements performed in the present study

Linear measurements	Angular measurement
Maxillary length (PNS-A): the distance between the posterior nasal spine (PNS) and point A	SNA: the angle formed between points S, N, and A, indicating the anteroposterior projection of the maxilla
Mandibular body length (Go L/R–Me): the distance between left and right gonion (Go) and menton (Me)	SNB: the angle formed between points S, N, and B, indicating the anteroposterior projection of the mandible
Anterior cranial fossa length (S–N): the distance between sella (S) and nasion (N)	ANB: the angle formed between points A, N, and B, indicating the anteroposterior intermaxillary relationship. In 3D analysis, unlike traditional cephalometrics, the difference between SNA and SNB could differ from the value of ANB
Total anterior facial height (N–Me): the distance between N and Me	Maxillomandibular (intermaxillary) angles (PNS–ANS–Go R/L–Me): the angles between the palatal and mandibular planes
Upper anterior facial height (N–ANS): the distance between N and the anterior nasal spine (ANS)	Total gonial angle (Cd R/L–Go R/L–Me): the angle between the mandibular ramus and body
Lower anterior facial height (ANS–Me): the distance between ANS and Me	Cranial base angle (Ba–S–N): the angle between basion (Ba), S, and N
Posterior facial height (Go L/R–S): the distance between S and left and right Go	Craniomaxillary angle (SN–PNS–ANS): the angle between the floor of the anterior cranial fossa and the palatal plane
Mandibular ramus height (Go L/R–Cd L/R): the distance between left and right condylion (Cd) and Go	Craniomandibular angle (SN–LGo–Me/SN–RGo–Me): the angle between the floor of the anterior cranial fossa and the mandibular plane, measuring mandibular divergence
Upper incisor midline and lower incisor midline to sagittal plane (UI–Sag P, LI–Sag P): the distances between the superior and inferior dental midlines and sagittal plane	

### Statistical analysis

Statistical analysis was performed using SPSS® v. 20.00 for Windows (IBM Corporation, Sommers, NY).

Intra- and interobserver agreement was analyzed by intraclass correlation coefficient (ICC) for all measurements. To evaluate and compare the precision of the measurements, the coefficient of variation (CoeffVar = SD/mean) was separately calculated for all cephalometric measurements. Means and SD were based on the four linear measurement values (two measurements by each of the two observers). The Shapiro-Wilk test was used to assess whether the data were normally distributed.

Differences in the mean CoeffVar between CBCT and MRI were evaluated using a two-sample *t* test. A Bland-Altman analysis [29] was used to assess the agreement between the two modalities with 95% limits of agreement. The ranges for the 95% limits of agreement (upper to lower) were supplied. All values (two measurements from each observer) were used in the assessment of agreement between the two imaging methods. Paired *t* tests were performed to compare CBCT measurements with the homologous MRI measurements.

*P* values less than 0.05 were considered statistically significant.

#### Sample size calculation

Sample size was calculated a posteriori, and retrospective power was established by G*Power (version 3.1.9.4, Franz Faul, Universita ¨t Kiel, Kiel, Germany) after data were collected due to the difficulty of finding patients with both tests (MRI, CBCT).

The values of the mean differences in the A-nasion-B (ANB) angle between the two modalities were used to perform the power analysis calculation along with the corresponding standard deviations. The data used to perform the analysis were mean difference ANB = 0.46, σ = 0.91, α = 0.05, and δ = 80.

The results of the power analysis indicated that to reach 80% power, 33 subjects per sample were necessary to perform the study.

Since ours is a pilot study and it cannot be used as a description of the power of this research, it can be legitimate to use to estimate the power and size of the sample for a future study. One of the most important reasons why a pilot study is needed is to obtain the required preliminary data for the calculation of a sample size for the primary outcome. The primary aim of pilot studies is not hypothesis testing; therefore, sample size is often not calculated. Some studies suggested for the pilot study over 30 samples per group [30], while some recommend 12 per group [31]. An appropriate sample size needs to be determined, not for providing appropriate power for hypothesis testing, but to understand the feasibility of participant recruitment or study design.

## Results

The measurement precision judged in terms of CoeffVar for all measurements showed equal precision for CBCT and MRI except for Ans-Me and Go R-Cd R, where MRI was significantly less precise (Table 2).

**Table 2:** Coefficient of variation, Intraobserver and interobserver agreement for three dimensional cephalometric measurements

Measurements	CoeffVar		Intraobserver ICC		Interobserver ICC^a^	
	CBCT Mean ± SD	MRI Mean ± SD	CBCT (Obs. I; Obs II)	MRI (Obs. I; Obs II)	CBCT	MRI
PNS-A (mm)	0.049 ± 0.002	0.036 ± 0.007	0.98; 0.96	0.94; 0.95	0.97	0.92
Go R–Me (mm)	0.029 ± 0.003	0.037 ± 0.015	0.99; 0.96	0.95; 0.97	0.98	0.80
Go L–Me (mm)	0.03 ± 0.001	0.031 ± 0.002	0.98; 0.95	0.87; 0.92	0.95	0.74
S–N (mm)	0.026 ± 0.007	0.029 ± 0.009	0.99; 0.90	0.81; 0.92	0.93	0.72
N–Me (mm)	0.048 ± 0.005	0.045 ± 0.008	0.98; 0.98	0.94; 0.97	0.99	0.87
N–ANS (mm)	0.049 ± 0.003	0.057 ± 0.002	0.98; 0.99	0.87; 0.90	0.98	0.92
ANS–Me (mm)	0.075 ± 0.004	0.083 ± 0.007	0.98; 0.97	0.91; 0.88	0.99	0.91
Go R–S (mm)	0.052 ± 0.003	0.054 ± 0.007	0.99; 0.99	0.90; 0.94	0.98	0.89
Go L–S (mm)	0.041 ± 0.006	0.044 ± 0.008	0.99; 0.99	0.92; 0.95	0.98	0.86
Go R–Cd R (mm)	0.019 ± 0.002	0.03 ± 0.01	0.97; 0.98	0.86; 0.95	0.94	0.89
Go L–Cd L (mm)	0.025 ± 0.004	0.021 ± 0.03	0.98; 0.95	0.78; 0.90	0.91	0.79
UI–Sag P (mm)	0.33 ± 0.15	0.38 ± 0.11	0.97; 0.99	0.83; 0.90	0.98	0.65
LI–Sag P (mm)	0.38 ± 0.06	0.40 ± 0.09	0.93; 0.96	0.75; 0.87	0.92	0.70
SNA	0.032 ± 0.002	0.031 ± 0.01	0.99; 0.98	0.76; 0.81	0.99	0.78
SNB	0.038 ± 0.003	0.032 ± 0.004	0.99; 0.98	0.72; 0.69	0.93	0.74
ANB	0.87 ± 0.25	0.97 ± 0.63	0.93; 0.98	0.85; 0.83	0.97	0.82
PNS–ANS–Go R–Me	0.076 ± 0.003	0.087 ± 0.02	0.99; 0.99	0.93; 0.95	0.97	0.84
PNS–ANS–Go L–Me	0.096 ± 0.004	0.097 ± 0.02	0.99; 0.98	0.92; 0.87	0.98	0.88
Cd R–Go R–Me	0.022 ± 0.005	0.026 ± 0.007	0.98; 0.98	0.93; 0.95	0.96	0.97
Cd L–Go L–Me	0.019 ± 0.003	0.022 ± 0.008	0.99; 0.98	0.92; 0.96	0.99	0.83
Ba–S–N	0.026 ± 0.003	0.028 ± 0.007	0.98; 0.99	0.94; 0.95	0.97	0.85
SN–PNS–ANS	0.30 ± 0.006	0.31 ± 0.04	0.98; 0.99	0.92; 0.94	0.98	0.82
SN–Go R–Me	0.076 ± 0.007	0.079 ± 0.005	0.96; 0.97	0.93; 0.95	0.98	0.92
SN–Go L–Me	0.085 ± 0.03	0.097 ± 0.07	0.97; 0.92	0.98; 0.97	0.96	0.89

*CoeffVar* coefficient of variation, *ICC* intraclass correlation coefficient, *Obs* observer

^a^Interobeserver ICC data are given by means of measurements of two time points and two investigators

**Fig. 3:**
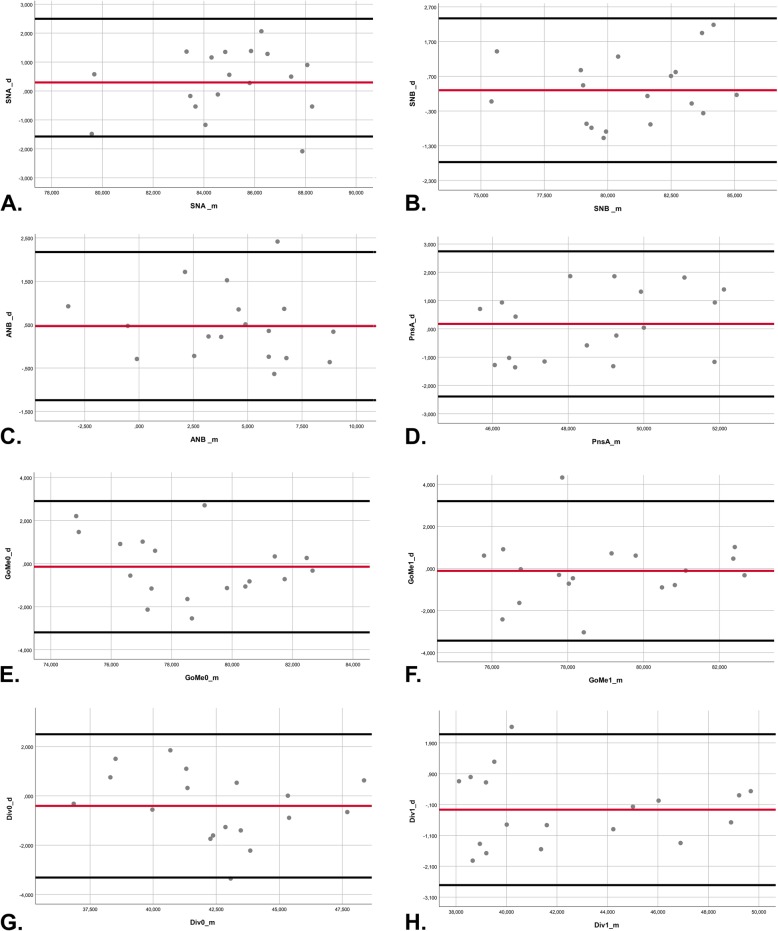
Bland-Altman plots show the differences between the measurements on CBCT and 3T-MRI. Red lines represent the mean of all differences (bias), and black lines represent the 95% limits of agreement. Exemplary measurements: **a** SNA angle, **b** SNB angle, **c** ANB angle, **d** PNS-A distance, **e** Go R-Me distance, **f** Go R-Me distance, **g** intermaxillary divergence R angle, and **h** intermaxillary divergence L angle

The intraobserver reliability (Table 2) for both observers showed high agreement for CBCT measurements. The average (± SD, range) intraobserver ICCs were 0.977 (± 0.016, 0.970–0.984) for observer I and 0.971 (± 0.022, 0.961–0.981) for observer II. High intraobserver ICCs were observed for the MRI even if slightly inferior, with mean values (± SD, range) of 0.881 (± 0.071, 0.849–0.91) for observer I and 0.912 (± 0.064, 0.884–0.939) for observer II. Interobserver reliability (Table 2) was gradely for CBCT with an average (± SD, range) ICC of 0.957 (± 0.032, 0.944–0.971). In comparison, interobserver reliability for MRI was also high but reasonably lower compared to CBCT, with an average ICC of 0.833 (± 0.08, 0.798–0.868). Bland-Altman analysis evinced high levels of agreement between the two modalities for all measurements. Bias range (mean ± SD) was − 0.25 to 0.66 mm (0.174 ± 0.31) for linear and − 0.41 to 0.54° (0.12 ± 0.33) for angular measurements (Table 3). Exemplary Bland-Altman plots of skeletal class according to Steiner [32], mandibular body length, maxillary length, and intermaxillary angles are shown in Fig. 3. No statistically significant difference was found between CBCT and MRI for all measurements (Table 3).

**Table 3:** Three dimensional cephalometric measurements from CBCT and MRI

Measurements	CBCT^a^	MRI^a^	Mean difference (CBCT-MRI)	95% limits of agreement	Significance
PNS-A (mm)	48.87 ± 2.38	48.69 ± 1.74	0.18	− 2.39; 2.74	NS
Go R–Me (mm)	78.41 ± 2.31	78.56 ± 2.96	− 0.14	− 3.19; 2.90	NS
Go L–Me (mm)	78.93 ± 2.43	79.05 ± 2.47	−0.12	− 3.43; 3.20	NS
S–N (mm)	65.85 ± 1.72	65.19 ± 1.89	0.66	− 1.56; 2.88	NS
N–Me (mm)	105.13 ± 5.06	105.36 ± 4.77	− 0.23	− 3.69; 3.24	NS
N–ANS (mm)	46.68 ± 2.30	46.93 ± 2.70	− 0.25	− 1.62; 1.11	NS
ANS–Me (mm)	60.44 ± 4.56	62.22 ± 5.22	0.22	− 2.69; 3.12	NS
Go R–S (mm)	81.78 ± 4.27	81.86 ± 4.39	− 0.08	− 3.20; 3.05	NS
Go L–S (mm)	82.21 ± 3.45	81.65 ± 3.59	0.45	− 2.38; 3.28	NS
Go R–Cd R (mm)	52.31 ± 1.02	51.78 ± 1.59	0.53	− 1.99; 3.04	NS
Go L–Cd L (mm)	51.92 ± 1.33	51.45 ± 1.11	0.47	− 1.57; 2.50	NS
UI–Sag P (mm)	3.27 ± 1.11	2.95 ± 1.14	0.32	− 2.58; 3.22	NS
LI–Sag P (mm)	2.31 ± 0.87	2.05 ± 0.83	0.26	− 0.99; 1.51	NS
SNA	85.09 ± 2.73	84.74 ± 2.70	0.46	− 1.57; 2.50	NS
SNB	81.10 ± 3.11	80.80 ± 2.61	0.30	− 1.77; 2.37	NS
ANB	4.32 ± 3.76	3.86 ± 3.78	0.47	− 1.24; 2.17	NS
PNS–ANS–Go R–Me	41.90 ± 3.22	42.30 ± 3.65	− 0.41	− 3.31; 2.50	NS
PNS–ANS–Go L–Me	42.39 ± 4.11	42.65 ± 4.16	− 0.27	− 2.71; 2.18	NS
Cd R–Go R–Me	117.88 ± 2.66	117.74 ± 3.11	0.13	− 2.04; 2.30	NS
Cd L–Go L–Me	118.43 ± 2.26	118.22 ± 2.57	0.21	− 1.18; 1.60	NS
Ba–S–N	127.56 ± 3.33	127.47 ± 3.56	0.03	− 2.47; 2.52	NS
SN–PNS–ANS	9.29 ± 2.78	8.75 ± 2.75	0.54	− 1.98; 3.06	NS
SN–Go R–Me	45.27 ± 3.47	45.48 ± 3.60	0.21	− 1.56; 1.99	NS
SN–Go L–Me	44.74 ± 3.82	45.07 ± 4.35	− 0.33	− 2.78; 2.13	NS

*NS* not significant

^a^Numerical data are given as means and standard deviations of measurements of two time points and two investigators

## Discussion

MRI seems to have a promising future in dentistry. It is a noninvasive and radiation-free examination, and after recent technological development, it has improved its image quality in mineralized tissues. MRI is now commonly used in TMD diagnosis, soft tissue pathologies, and bone topography for the placement of dental implants [33, 34].

The aim of this study was to determine the validity of MRI as a support for three-dimensional analysis for treatment planning in orthodontics. The results of this preliminary study seem to support the possible use of 3T-MRI as a reliable and accurate alternative to CBCT in three-dimensional evaluation of malocclusion. No other three-dimensional cephalometric tracing on MRI, to the authors’ knowledge, has been evaluated before.

A T2-weighted sequence with good contrast, high spatial resolution, and short scanning time seemed to the authors to be the best option as many orthodontic patients are children. MRI images on cephalometric software allow in most cases a clear visualization of dental and skeletal cephalometric landmarks. High levels of agreement between the measurements on CBCT and the corresponding measurements on 3T-MRI have been found by examining adult patients. No statistically significant difference between the two modalities was found for all linear and angular measurements. The mean difference in Bland-Altman still indicated a low and seemingly clinically acceptable bias for all measurements.

However, intra- and interobserver ICC in 3T-MRI was not as good as in CBCT scans, possibly due to less experience of operators in determining cephalometric landmarks on magnetic resonance scans. Reduced sample size could also easily underestimate or overestimate these values, as little changes in the position of cephalometric points may lead to great variations of ICC. This variability could also be attributed to the different details over mineralized structures in MRI whose quality mainly depends on two factors: immobility during scanning and noise (the set of unwanted interferences that overlap the main transmission by corrupting or submerging it in an additive way).

The absence of ionizing radiation as the main advantage in the use of magnetic resonance is particularly significant in disciplines such as orthodontics, where most patients are young [35]. Therefore, MRI could be a valuable tool to analyze both soft tissues and hard tissue for orthodontic diagnosis/treatment planning purposes.

Considering the overall good concordance with CBCT (gold standard for bony measurements) and the absence of radiation exposure, 3T-MRI cephalometric analysis for the evaluation and monitoring of orthodontic and orthognathic conditions could be performed in the future to reduce radiation dose, which is crucial in young patients, and overcome the bidimensional limits of lateral cephalometric analysis.

Among the main limitations of MRI, it should be named claustrophobia of some patients, difficulty of maintaining a still position especially in younger patients, and difficulties in performing the examination when a metal appliance is worn by the patient, thus making this examination less likely to be performed as an interim evaluation of orthodontic treatment. Possible solutions to these problems may be open gantry MRI, adopting shorter scanning protocols compatible with clinically acceptable definitions, and the use of devices built with nonmagnetic materials, such as ceramic brackets and titanium appliances [36]. Other limitations of MRI are costs and machine availability. Hopefully as happened for CBCT, we will assist in a continuous improvement in the performance of the machines and a progressive cost reduction. Three Tesla MRI scans would need to be read by medical radiologists to identify incidental (hard and soft tissue) findings in orthodontic patients. Furthermore, the fee for medical radiologists would in turn add more cost to orthodontic care.

The MRI scanning protocol used in all patients included in this study appears to provide a definition acceptable for landmark identification and a total scan time of less than 6 min, thus making it less likely to have motion artifacts in adult patients.

High-field magnets could also give a substantial improvement in the signal-to-noise ratio (SNR), making it possible to identify better cephalometric landmarks.

A three-dimensional cephalometric analysis could lead to more precise and conclusive diagnoses compared to two-dimensional radiographs. Many proposals in CBCT-based 3D-cephalometry have been published, but no evidence-based procedure could be established due to the absence of comparative norms [37]. 3T-MRI because of its lower biologic cost could provide the possibility to establish proper standards for 3D cephalometry, as all orthodontic conditions, including normal collectives and patients without severe malocclusions, could be analyzed. Taking into consideration the actual costs and benefits of cephalometric studies performed on 3T-MRI, it is the authors’ opinion that bidimensional lateral cephalogram and orthopantomography would serve orthodontists well, at least in the near future, with low dosage limited view CBCT as an option for complex cases, such as supernumerary or agenesic teeth, canine impaction, or digital planning of temporary anchorage devices insertion [11].

Although these values resulted from a small sample, they seem to suggest how 3T-MRI technology can have clinical applications in orthodontic diagnosis in the future, as other studies already have been proven in other fields of dentistry, such as implantology [34].

## Conclusion

In orthodontics, it is often advisable to obtain as many three-dimensional information as possible, but the biologic cost of second-level radiologic examinations does make it not worthy. In this preliminary analysis, it appears that precise and accurate 3D cephalometric analysis can be obtained. The proposed method appears to be, albeit with a lower performance compared to CBCT, sufficiently precise and reliable for clinical purposes.

The results of this study indicate that 3D cephalometric analysis on 3T-MRI is an interesting topic to be further investigated in the future with the potential to become a routine application for orthodontic treatment planning with the advantage of MRI in the visualization of soft tissues. This could have an important effect on treatment planning and monitoring in orthodontic and orthognathic patients, especially if young, because MRI can be repeated and has apparently no biologic costs.

Further studies with larger samples should be conducted to support our findings and to assess whether MRI could be used for 3D cephalometric analysis instead of CBCT.

## Data Availability

The data that support the findings of this study are available from Fondazione IRCCS Cà Granda Ospedale Maggiore Policlinico Milan (Italy), but restrictions apply to the availability of these data, which were used under license for the current study, and so are not publicly available. Data are, however, available from the authors upon reasonable request and with permission of Fondazione IRCCS Cà Granda Ospedale Maggiore Policlinico Milan (Italy).

## Abbreviations

3T-MRI: 3-T high-field MRI
3D: Three dimensional
CBCT: Cone-beam computerized tomography
MRI: Magnetic resonance imaging
